# Assessment of Human Factors After Advanced Life Support Courses Comparing Simulated Team and Real Team Assessment: A Randomized Controlled Cohort Trial

**DOI:** 10.3389/fcvm.2022.840114

**Published:** 2022-07-15

**Authors:** Sabine Nabecker, Sören Huwendiek, Christian Seidl, Anisa Hana, Lorenz Theiler, Robert Greif

**Affiliations:** ^1^Department of Anesthesiology and Pain Medicine, Bern University Hospital, University of Bern, Bern, Switzerland; ^2^Department of Anesthesiology and Pain Management, Sinai Health System, University of Toronto, Toronto, ON, Canada; ^3^ERC Research NET, Niel, Belgium; ^4^Graduate School for Health Sciences (GHS), University of Bern, Bern, Switzerland; ^5^Department for Assessment and Evaluation, Institute for Medical Education, University of Bern, Bern, Switzerland; ^6^EMS Rescue Service of the Canton Basel-Stadt, Basel, Switzerland; ^7^Department of Intensive Care Medicine, Laurentius Hospital, Roermond, Netherlands; ^8^Department of Anesthesia, Kantonsspital Aarau, Aarau, Switzerland; ^9^School of Medicine, Sigmund Freud University Vienna, Vienna, Austria

**Keywords:** education, CPR, life support, European Resuscitation Council (ERC), human factors

## Abstract

**Aim:**

Human factors are essential for high-quality resuscitation team collaboration and are, therefore, taught in international advanced life support courses, but their assessment differs widely. In Europe, the summative life support course assessment tests mainly adhere to guidelines but few human factors. This randomized controlled simulation trial investigated instructors’ and course participants’ perceptions of human factors assessment after two different summative assessments.

**Methods:**

All 5th/6th-year medical students who attended 19 advanced life support courses according to the 2015 European Resuscitation Council guidelines during one study year were invited to participate. Each course was randomized to either: (1) Simulated team assessment (one instructor simulates a team, and the assessed person leads this “team” through a cardiac-arrest scenario test); (2) Real team assessment (4 students form a team, one of them is assessed as the team leader; team members are not assessed and act only on team leader’s commands). After the summative assessments, instructors, and students rated the tests’ ability to assess human factors using a visual analog scale (VAS, 0 = no agreement, 10 = total agreement).

**Results:**

A total of 227 students participated in the 1-day Immediate Life Support courses, 196 students in the 2-day Advanced Life Support courses, additionally 54 instructors were included. Instructors judged all human factors significantly better in real team assessments; students rated leadership and situational awareness comparable between both assessments. Assessment pass rates were comparable between groups.

**Conclusion:**

Summative assessment in real teams was perceived significantly better to assess human factors. These results might influence current summative assessment practices in advanced life support courses.

## Introduction

The incidence of cardiac arrests ranges between one and five cardiac arrests per 1,000 hospital admissions (1), and 67–170 per 1,000,000 citizens (2). Cardiopulmonary resuscitation (CPR) is a key skill that all health care professionals should master in order to improve patient outcomes after cardiac arrests. Especially faster and more efficient in-hospital cardiopulmonary resuscitation and early defibrillation can improve patient outcomes significantly (1–3).

All health care professionals should be proficient in basic life support measures (4, 5) and should be regularly trained in small groups with a maximum instructor to participant ratio of 1:6 (6). Health care professionals who regularly treat cardiac arrests need to participate in Advanced Life Support (ALS) courses to improve their patients’ outcomes (7, 8). Those health care professionals who are infrequently involved in cardiac arrest management should at least participate in Immediate Life Support (ILS) courses (4, 9).

During a cardiac arrest, physicians usually assume the role of the team leader and need to be proficient in guiding an interdisciplinary resuscitation team through the management of a cardiac arrest. The 2015 and 2021 Guidelines of the European Resuscitation Council (ERC) emphasize that team membership and leadership training are essential (5, 9–11).

Prerequisites for proper team performance were identified earlier, and include: team leadership, task management, teamwork, situational awareness, decision-making, adaptability, event and mission analysis, and communication (12, 13). ERC ALS courses include training of technical skills, adherence to current resuscitation guidelines, and training of human factors focusing on team membership and leadership, task management, communication, and situational awareness (14).

Different approaches to assessing human factors in different fields were described (e.g., the ANTS framework, the Oxford NOTECHS, the Mayo High performance teamwork scale, the adapted LOSA checklist from aviation, the Queen’s Simulation Assessment Tool, etc.) (12, 15–18). The paradigm shift to also include human factor training in cardiopulmonary resuscitation courses took place over the last years, however, the practical end-of-course summative assessment has remained largely unchanged and tests predominantly the participants’ ability to adhere to current resuscitation guidelines. Currently, the ERC uses simulated team assessments of one candidate during the summative practical end-of-course assessment. One instructor simulates a team, the assessed person leads this “team” through a cardiac arrest scenario test. Other organizations like the American Heart Association use real team assessments, where a group of participants forms a team. One of them is then assessed as the team leader; the team members are usually not assessed and act only on the commands of the team leader. There is currently no evidence available as to what extent different assessment methods can test human factors.

Therefore, the aim of this study was to investigate which variant of these two different summative end-of-course assessment methods is perceived as being superior in their ability to assess human factors, as judged by instructors and students. This is an evaluation of the opinions of course participants and instructors, the null hypothesis was that there is no difference between leadership skills comparing both assessment methods. The results of this study might influence the development of a different assessment approach in such cardiopulmonary resuscitation courses.

## Materials and Methods

### Participants and Ethics

After reviewing the protocol, the Cantonal Ethics Committee of Bern, Switzerland (Req-2017-00579, 07.08.2017) judged the study as non-human research according to the Swiss Human Research Act. After registration at clinicaltrials.gov (NCT 03381443), this randomized controlled simulation trial was performed at the University of Bern, Switzerland between December 2017 and March 2019.

All 5th-year medical students in the study year 2017/2018 took the mandatory 8-h ILS course; all 6th-year medical students the 16-h ALS course. These courses followed the 2015 ERC course structure and students earned an internationally valid certification (9). All course instructors were ERC certified ILS/ALS instructors. With written informed consent from all participants and instructors, all assessments were video recorded. Course participants participated only once, whereas most instructors participated more than once.

### Procedures

The advanced life support courses ended with a mandatory summative assessment that used four different, validated ERC cardiac arrest scenario tests (19). All courses were randomized to one of two different assessment methods:

1. Simulated team assessment: One instructor mimes a whole team, and the assessed course participant acts as team leader, leading this “team” through a cardiac arrest scenario test. This is the current standard ERC ILS/ALS course assessment.
2. Real team assessment: Four course participants are together in the assessment room and form a team. However, only the predefined team leader is assessed, all other team members act as resuscitation team, but they are not allowed to help the team leader with medical decisions and act only on commands of the assessed team leader. This corresponds to the American Heart Association Advanced Cardiac Life Support course assessment.

### Randomization

All participants were scheduled for their respective courses by the University of Bern, the study team had no influence on which participant attended which course. Due to the study design, each course was randomized, not the individual participant. Randomization was performed using the online software “Research Randomizer^1^.”

### Data Analysis

Participants provided general characteristics including age, gender, primary language, experience as an instructor or real-life CPR experience, number of courses taught, and registration as Bern First Responder.

After the assessment, all course participants answered a questionnaire on their opinion to what extent the assessment was able to assess human factors such as team leadership, team membership, communication skills, team management skills, and situational awareness. They rated their self-assessed level of competence as a team member and team leader and judged their need for further training as a team member or team leader. In addition, they were asked to judge the assessment’s ability to test medical resuscitation knowledge and technical CPR-skills. All measurements used a visual analog scale (VAS) ranging from 0 to 10, where 0 meant no agreement and 10 meant total agreement. Participation in the study was voluntary and had no effect on the medical students’ grading at the university (20). All participating ILS/ALS instructors were also asked to fill out the same questionnaire, once after each assessment day, not after each individual assessment. The primary outcome parameter is the difference in team leadership assessment in participants’ and instructors’ opinions.

After 1 year, participants of the former ILS-course were asked to fill out a follow-up questionnaire before they took their 6th-year ALS-course. Participants of the original ALS-course could not take part in the follow-up, because they had already graduated from university and were not accessible to the study team.

For pass or fail ratings of the assessments, the official ERC course-grading scheme on the official ERC ALS scenario test assessment forms was used. Participants are judged for each parameter on a scale from 1 to 4 (1 = outstanding, 2 = adequate, 3 = marginal, 4 = insufficient).

### Statistics

No formal sample size calculation was performed as we did not find any literature to base such a calculation on. All students of the entire study year taking these advanced life support courses were included.

Statistical analysis was performed using the software STATA version 16.0 (StataCorp LT, TX, United States). Descriptive statistics analyzed participants’ characteristics by using either a *t*-test or Chi-square test as applicable. Questionnaire results were non-parametric and therefore Mann–Whitney-*U* test was used to evaluate them. Assessment sub-items were evaluated using Chi-square tests. Data are presented as value (percentage) or mean ± SD (95% CI)—we opted to visualize our data with mean ± SD to allow for better comparability as the data was not that skewed compared to the median (interquartile range). We considered a two-sided *p*-value < 0.05 as statistically significant.

## Results

**FIGURE 1:**
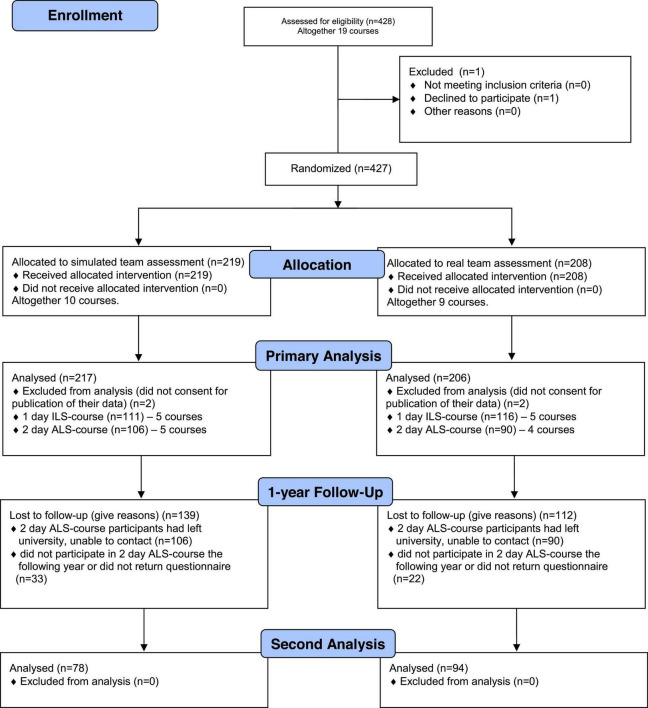
Consort flow diagram.

A total of 427 medical students participated in the study (in 19 courses), and one declined participation. Figure 1 shows the Consort Flow Diagram. Four students had to be excluded from the analysis because they withdrew their consent to publication after participating in the advanced life support course and assessment. In the 1-day ILS-course, 111 students were randomized to the simulated team assessment, and 116 to the real team assessment (5 courses each). In the 2-day ALS-course, 106 students to the simulated team assessment (5 courses), and 90 to the real team assessment (4 courses). The 1-year follow-up reached 172 students, 78 from the simulated team assessment and 94 from the real team assessment.

A total of 54 instructors participated in the study; they were aged 39 ± 8 years; 17 (31%) were women; the primary language reported was: 51 German (94%), 2 French (4%), and 1 (2%) other; mean experience as the instructor was 4 ± 4 years; courses taught before participating in the study: 3 ± 2; 17 (31%) were registered Bern First Responders. Most instructors participated more than once.

Participating instructors judged the real team assessment overall significantly better than the simulated team assessment (*p* = 0.029, Table 1). All human factors were judged significantly better in the real team assessment (*p* < 0.01 to 0.024), and so was the ability to test CPR skills (*p* < 0.01). In contrast, knowledge could be tested equally well with both assessment methods (*p* = 0.842, Table 1).

**TABLE 1:** Usefulness of the assessment method to assess human factors as perceived by the instructor.

Characteristic/Assessment	Simulated team	Real team	*P-value* ^a^
	(*n* = 49)	(*n* = 40)	
Team leader	5.6 ± 2.4	7.2 ± 2.5	< 0.01
	(5.0–6.3)	(6.5–8.0)	
Team member	1.9 ± 2.2	3.6 ± 2.9	< 0.01
	(1.3–2.6)	(2.7–4.5)	
Communication	3.9 ± 2.7	6.1 ± 3.0	< 0.01
	(3.1–4.7)	(5.2–7.1)	
Team management	3.7 ± 3.0	6.7 ± 2.5	< 0.01
	(2.8–4.6)	(5.9–7.5)	
CPR skills	3.4 ± 3.2	5.9 ± 3.2	< 0.01
	(2.5–4.3)	(4.9–6.9)	
Knowledge	7.2 ± 1.8	7.1 ± 2.2	0.842
	(6.7–7.7)	(6.4–7.8)	
Situational awareness	6.1 ± 2.4	7.1 ± 2.2	0.024
	(5.4–6.8)	(6.4–7.8)	
Assessment overall	5.7 ± 1.9	6.5 ± 2.7	0.029
	(5.2–6.3)	(5.6–7.3)	

*Data are mean ± SD (95% CI).Values: VAS, Visual Analog Scale (0–10).*

*^a^Mann–Whitney-U test. Altogether 54 instructors participated in the study, some instructors participated more than once. This table shows the answers from each instructor after 1 day of assessments, not after each individual assessment.*

Table 2 shows details of the participating students’ characteristics. Of note, even though courses were randomized, we found in the 2-day ALS course cohort in the real team assessment group significantly more women (67% vs. 38% in the simulated team assessment group, *p* < 0.01). Also regardless of randomization, more registered Bern First Responders were found in the 2-day ALS course assessed with the simulated team assessment method (15% vs. 2% for team assessment, *p* < 0.01).

**TABLE 2:** Course participants’ characteristics.

Characteristic	1-day ILS-course			2-day ALS-course		
Assessment	Simulated team	Real team		Simulated team	Real team	
	*n* = 111	*n* = 116	*P-value*	*n* = 106	*n* = 90	*P-value*
Age, years	24.5 ± 1.7	24.6 ± 2.7	0.833^a^	26.0 ± 1.8	25.6 ± 1.9	0.111^a^
	(24.2–24.9)	(24.1–25.1)		(25.7–26.4)	(25.2–26.0)	
Female	72 (65)	69 (59)	0.403^b^	40 (38)	60 (67)	< 0.01^b^
Primary language						
- German	97 (87)	96 (83)	0.342^b^	91 (86)	85 (95)	0.112^b^
- French	9 (8)	9 (8)		6 (6)	3 (3)	
- Other	5 (5)	11 (9)		9 (8)	2 (2)	
Real life CPR experience	5 (5)	6 (5)	0.803^b^	31 (29)	23 (26)	0.564^b^
Bern First Responder	16 (14)	22 (19)	0.359^b^	16 (15)	2 (2)	< 0.01^b^

*^a^t-test, ^b^Chi-square test. ILS, Immediate Life Support; ALS, Advanced Life Support; CPR, cardiopulmonary resuscitation. A First Responder is a trained layperson or healthcare professional who is dispatched via an app to a medical emergency in addition to an ambulance. Data are value (percentage) or mean ± SD (95% CI).*

After 1 year, in the ILS cohort, 22 students reported having resuscitated a patient in real life after passing the last year’s ILS-course (15 in the simulated team assessment group, 7 in the real team assessment group). Additional 11 students were recruited as Bern First Responders (4 in the simulated team assessment group and 7 in the real team assessment group).

Details of the students’ subjective ratings of their assessments are displayed in Table 3. Self-assessed competence as a team member was judged significantly better for the real team assessment (ILS-course *p* = 0.032; ALS-course *p* < 0.01). Real team assessment was judged consistently significantly better in the ability to assess team membership, communication skills, team management skills, and CPR skills (all *p* < 0.01). Situational awareness was judged only in the ILS-course cohort in favor of real team assessments (*p* = 0.023, Table 3).

**TABLE 3:** Course participants self-evaluation and usefulness of the assessment method to assess human factors as perceived by the course participants.

Characteristic	1-day ILS-course			2-day ALS-course		
Assessment	Simulated team	Real team		Simulated team	Real team	
	*n* = 111	*n* = 116	*P-value* ^a^	*n* = 106	*n* = 90	*P-value* ^a^
Competent as team member	7.1 ± 1.6	7.6 ± 1.2	0.032	7.2 ± 1.7	7.8 ± 1.5	<0.01
	(6.8–7.4)	(7.4–7.9)		(6.9–7.6)	(7.5–8.1)	
Further training as team member needed	6.4 ± 2.3	5.8 ± 2.9	0.189	6.8 ± 2.6	5.5 ± 3.1	<0.01
	(6.0–6.8)	(5.3–6.4)		(6.3–7.3)	(4.9–6.2)	
Competent as team leader	5.8 ± 1.8	6.0 ± 1.8	0.422	5.7 ± 2.2	6.0 ± 1.9	0.286
	(5.4–6.1)	(5.7–6.3)		(5.3–6.1)	(5.6–6.4)	
Further training as team leader needed	8.4 ± 1.6	8.2 ± 1.8	0.791	8.7 ± 1.5	8.1 ± 2.0	0.064
	(8.1–8.7)	(7.9–8.5)		(8.4–8.9)	(7.7–8.5)	
Team leader	8.1 ± 1.8	8.5 ± 1.2	0.327	7.5 ± 2.3	8.0 ± 2.0	0.145
	(7.7–8.4)	(8.3–8.7)		(7.1–8.0)	(7.6–8.4)	
Team member	4.2 ± 3.4	6.0 ± 2.9	<0.01	4.0 ± 3.3	5.3 ± 3.3	<0.01
	(3.5–4.8)	(5.5–6.6)		(3.4–4.6)	(4.6–6.0)	
Communication	6.7 ± 2.3	7.6 ± 1.8	<0.01	5.9 ± 3.0	7.4 ± 2.5	<0.01
	(6.3–7.1)	(7.3–8.0)		(5.3–6.5)	(6.9–7.9)	
Team management	6.4 ± 2.6	8.1 ± 1.6	<0.01	5.7 ± 3.1	7.7 ± 2.3	<0.01
	(5.9–6.9)	(7.8–8.4)		(5.1–6.3)	(7.2–8.2)	
CPR skills	5.9 ± 3.3	7.8 ± 2.0	<0.01	5.8 ± 3.4	7.1 ± 2.6	<0.01
	(5.3–6.6)	(7.5–8.2)		(5.1–6.5)	(6.6–7.7)	
Knowledge	7.0 ± 2.0	7.1 ± 1.9	0.734	7.8 ± 1.5	7.6 ± 1.9	0.585
	(6.6–7.3)	(6.8–7.5)		(7.6–8.1)	(7.2–8.0)	
Situational awareness	7.8 ± 1.6	8.2 ± 1.6	0.023	8.0 ± 1.9	8.0 ± 2.2	0.673
	(7.5–8.1)	(7.9–8.5)		(7.7–8.4)	(7.6–8.5)	
Assessment overall	7.9 ± 1.8	8.2 ± 1.4	0.515	7.5 ± 2.3	7.5 ± 2.1	0.756
	(7.6–8.3)	(8.0–8.5)		(7.0–7.9)	(7.0–7.9)	

*Data are mean ± SD (95% CI). ILS, Immediate Life Support; ALS, Advanced Life Support; VAS, Visual Analog Scale (0–10); CPR, cardiopulmonary resuscitation.*

*^a^Mann–Whitney-U test.*

In the 1-year follow-up, both the self-assessed competence as a team member and team leader were comparable (*p* = 0.194 and 0.372). The team member and team leader competences dropped for both assessment methods (all *p* < 0.01). All students agreed at the time of the follow-up that further training is necessary for team members and leaders (all *p* > 0.05).

Table 4 shows the detailed results of the assessments. ILS course participants had a first attempt cardiac-arrest simulation-test passing rate of 80–84%; whereas ALS course participants in 92-95%. The detailed results are comparable between both course types and assessments, but some items scored differently (Table 4).

**TABLE 4:** Objective results from the official European Resuscitation Council cardiac arrest scenario teaching forms as judged by the instructors.

Characteristic	1-day ILS course			2-day ALS course		
Assessment	Simulated team	Real team	*P-value* ^a^	Simulated Team	Real team	*P-value* ^a^
	*n* = 111	*n* = 116		*n* = 106	*n* = 90	
**Overall Result**						
First attempt successful	93 (84)	93 (80)	0.480	101 (95)	83 (92)	0.373
**Detailed Results**						
ABCDE*	17/49/18/4	17/54/39/1	0.065	57/34/12/1	51/28/7/0	0.673
	(19/56/20/5)	(15/49/35/1)		(55/33/11/1)	(59/33/8/0)	
Oxygen and vascular access*	28/45/16/2	29/76/2/0	< 0.01	58/43/4/0	55/31/3/0	0.652
	(31/49/18/2)	(27/71/2/0)		(55/41/4/0)	(62/35/3/0)	
Recognizes condition*	28/36/14/11	14/52/16/13	0.053	69/27/8/1	58/20/6/0	0.811
	(31/41/16/12)	(15/54/17/14)		(65/26/8/1)	(69/24/7/0)	
Gives medication*	18/31/10/21	20/27/8/33	0.408	60/27/13/1	62/15/6/2	0.169
	(22/39/13/26)	(23/31/9/37)		(59/27/13/1)	(73/18/7/2)	
Further medication*	7/18/4/35	8/23/8/33	0.659	33/34/15/5	29/21/12/4	0.823
	(11/28/6/55)	(11/32/11/46)		(38/39/17/6)	(44/32/18/6)	
Other treatment*	2/15/2/38	7/12/9/39	0.078	28/24/18/5	31/20/11/7	0.514
	(4/26/4/66)	(10/18/14/58)		(37/32/24/7)	(45/29/16/10)	
Recognizes arrest*	65/33/8/3	61/48/6/1	0.264	76/25/4/0	67/17/6/0	0.511
	(60/30/7/3)	(53/41/5/1)		(72/24/4/0)	(74/19/7/0)	
Call for help*	44/29/10/20	38/48/8/8	0.015	30/38/16/4	43/25/6/0	< 0.01
	(43/28/10/19)	(37/47/8/8)		(34/43/18/5)	(58/34/8/0)	
CPR 2 min*	67/37/4/1	61/48/3/0	0.422	74/26/3/1	68/16/3/0	0.544
	(61/34/4/1)	(54/43/3/0)		(71/25/3/1)	(78/18/4/0)	
Airway*	57/47/3/0	45/62/2/1	0.201	65/30/2/1	69/16/2/1	0.278
	(53/44/3/0)	(41/56/2/1)		(66/31/2/1)	(79/18/2/1)	
Monitoring*	62/38/3/2	54/49/0/1	0.153	66/26/3/0	61/21/3/0	0.917
	(59/36/3/2)	(52/47/0/1)		(70/27/3/0)	(72/25/3/0)	
Epinephrine*	61/34/6/5	47/44/12/10	0.088	75/18/7/2	62/19/4/1	0.787
	(57/32/6/5)	(41/39/11/9)		(73/18/7/2)	(73/20/6/1)	
Reversible causes*	37/35/16/10	8/42/24/14	< 0.01	55/35/7/2	46/23/11/2	0.458
	(38/36/16/10)	(9/48/27/16)		(56/35/7/2)	(56/28/14/2)	
Recognizes rhythm change*	68/31/5/3	75/36/3/1	0.587	85/16/3/1	66/18/2/1	0.794
	(63/29/5/3)	(65/31/3/1)		(81/15/3/1)	(76/21/2/1)	
Defibrillation*	67/31/6/4	73/41/1/0	0.029	85/16/3/1	68/15/3/2	0.858
	(62/29/5/4)	(63/36/1/0)		(81/15/3/1)	(77/17/4/2)	
CPR 2 min*	68/35/2/0	70/41/2/1	0.769	79/22/2/0	68/15/1/1	0.621
	(65/33/2/0)	(61/36/2/1)		(77/21/2/0)	(80/18/1/1)	
Recognizes rhythm change*	64/30/3/3	61/41/1/2	0.414	83/16/2/1	59/17/1/1	0.743
	(64/30/3/3)	(58/39/1/2)		(81/16/2/1)	(76/22/1/1)	
Further Epinephrine*	40/31/6/15	26/37/10/9	0.142	34/26/23/3	34/22/8/7	0.050
	(43/34/7/16)	(32/45/12/11)		(40/30/27/3)	(48/31/11/10)	
Minimizes interruptions*	61/32/3/1	58/38/5/2	0.742	68/32/1/1	63/14/4/1	0.074
	(63/33/3/1)	(56/37/5/2)		(67/31/1/1)	(77/17/5/1)	
Defibrillation*	57/28/9/5	42/44/9/4	0.115	78/16/8/3	61/14/3/2	0.722
	(58/28/9/5)	(42/45/9/4)		(74/15/8/3)	(76/18/4/2)	
**Detailed Results**						
CPR 2 min*	65/29/2/1	53/37/6/0	0.159	74/23/3/0	61/16/1/1	0.557
	(67/30/2/1)	(55/39/6/0)		(74/23/3/0)	(77/21/1/1)	
Recognizes rhythm change*	58/30/1/2	48/45/5/0	0.039	76/24/2/0	65/18/1/1	0.679
	(64/33/1/2)	(49/46/5/0)		(75/23/2/0)	(77/21/1/1)	
Signs of life*	51/32/4/2	44/46/3/2	0.395	70/26/6/0	66/18/1/1	0.186
	(57/36/5/2)	(46/49/3/2)		(69/25/6/0)	(77/21/1/1)	
Post resuscitation care*	31/36/11/1	14/39/11/1	0.156	46/36/4/1	39/22/4/1	0.779
	(39/46/14/1)	(22/60/17/1)		(53/41/5/1)	(59/33/6/2)	

Data are value (percentage). ILS, Immediate Life Support; ALS, Advanced Life Support; CPR, cardiopulmonary resuscitation. Scale: 1 = outstanding, 2 = adequate, 3 = marginal, 4 = insufficient. ABCDE, Airway, Breathing, Circulation, Disability, Exposure/Environment. *Missing values, ^a^Chi-square test.

## Discussion

Instructors of advanced life support courses judge the real team assessment overall significantly better and rate its ability to test all human factors and CPR-skills better compared to the simulated team assessment. Participating students agree that team membership and management, communication, and CPR skills were better assessed by the real team assessment. However, students rated the assessments as comparable in the following categories: the assessment in general, team leadership, and knowledge. Situational awareness was only rated better in the 1-day ILS course, whereas judged as comparable in the 2-day ALS course.

Our course participants self-assessed their competence as team members higher than as team leaders, regardless of the assessment method used. They also agreed that further training as a team leader is more important than team membership training. These findings are consistent with findings from other researchers (21, 22).

The importance of training human factors in cardiopulmonary resuscitation courses is well established (23–25). Resuscitation teams who applied human factors efficiently also performed better in technical resuscitation skills (26). If a team leader performs hands-on procedures, the whole team is less effective (27). Prior participation in an advanced life support course improved the team leader’s ability to stay hands off (27). Nowadays, all courses include team leadership training, even though there is very little evidence that such specific leadership training during those courses leads to improved patient outcome (10). However, training in resuscitation skills, in general, was able to improve patient outcome (7).

It was unclear if current end-of-course summative assessments can test these human factor competences adequately. Therefore, the current study asked participants and instructors to judge subjectively two different assessment methods, the simulated team assessment, and the real team assessment. Participants found that both assessments are equally effective to test their team leadership skills. However, the included instructors who have experience with multiple course participants have a different opinions. The instructors clearly judge the real team assessment as superior in its ability to test team leadership skills. Training leadership skills prior to being involved in a real cardiac arrest is important (28, 29). Unfortunately, our data shows only the opinions of course participants and instructors. Further research is necessary to objectively assess the competences for such team leadership factors, and if one assessment approach is suitable to assess these competences in different advanced life support formats (e.g., for neonatal, pediatric, or adult resuscitation).

Participants of a qualitative analysis of human factors identified teamwork issues as most challenging during pediatric cardiac arrests (21). Our participants rated team membership competences consistently lower than team leadership competences. The real team assessment was judged to be better suitable to test team membership compared to the simulated team assessment. The lower scores for team membership compared to the other aspects of this study introduces the question if there is a need to adjust the current assessment method to allow better assessment of team membership competences. Regardless of the assessment method used, team members are not allowed to act as “real team members” during an assessment, they only act on team leaders’ commands. Assessment of team member competences is therefore not possible. In contrast, during cardiopulmonary resuscitation courses, team membership skills are trained, and course participants are encouraged to act as they would in real life and support their team leader with all their knowledge and skills.

Communication during a cardiac arrest needs to be clear, concise, brief, empathetic, and trigger a feedback loop. It needs to be targeted directly at a person and closing the loop is advisable (30, 31). ERC cardiopulmonary resuscitation courses emphasize proper communication and specifically focus on hindering factors for speaking up, which is key for success in cardiac arrest situations (29, 30). Research showed that simple and short ongoing educational interventions on leadership principles can improve team leadership and communication competences significantly (24, 32, 33). Our results show that such communication competences can be better assessed with the real team assessment compared to the simulated team assessment.

Shared situational awareness enables better teamwork (30). Both assessment methods were rated comparably by our ALS course participants. However, ILS course participants and instructors think situational awareness can be better assessed by real team assessments. The inconsistency in these findings suggests that situational awareness might depend more on the assessment setting itself, than on the assessment method.

Interestingly, over the 1 year between the initial study and the follow-up, 11 medical students (participants of the 1-day ILS course) were successfully recruited as Bern First Responders, some of them during the ILS courses (34) (A First Responder is a trained layperson or healthcare professional who is dispatched *via* an app to a medical emergency in addition to an ambulance). This should be considered a positive outcome given that health care professionals might get easily overwhelmed with the additional burden of being a First Responder (35).

This study has several limitations. It was a single-center study focusing on medical students as course participants in official European Resuscitation Council advanced life support courses. The University of Bern offers a large medical program with more than 340 medical students per study year. The medical students were randomly assigned by the University to their courses. There is a slight chance that medical students assessed together also worked together during course work or clinically as a team of students prior to their participation in the respective courses, therefore, we cannot exclude an effect of this on leadership and teamwork. Groups for the real team assessment were chosen randomly, and care was taken that students who had trained together during the course were not assessed together. Although there have been many study participants in this study, there are two cohorts of participants which limits the generalizability of the data, half of the study participants participated in a 1-day ILS course, and half in a 2-day ALS course. Randomization was performed for the whole course due to practical reasons and not for the individual course participant.

On the other hand, this study has several strengths as it was planned and executed as a large randomized controlled trial and each participant was only included once. Both the instructors’ and the participants’ perspectives are included.

In conclusion, instructors of advanced life support courses rated a summative course assessment in real teams significantly better regarding the ability to test team leadership, team membership, communication, team management, and situational awareness compared to simulated team assessments, which is the current European Resuscitation Council’s standard end-of-course assessment. Course participants rated team leadership and partly situational awareness as comparable between both assessment methods. These results might influence current summative assessment practices in advanced life support courses.

## Data Availability Statement

The original contributions presented in this study are included in the article/supplementary material, further inquiries can be directed to the corresponding author with a dedicated research question and local ethics committee approval.

## Ethics Statement

The studies involving human participants were reviewed and approved by Cantonal Ethics Committee of Bern, Switzerland. The patients/participants provided their written informed consent to participate in this study.

## Author Contributions

SN had the idea of the study, performed data acquisition, data evaluation, first manuscript, and approved final manuscript. SH and LT contributed to data evaluation, significant contribution to manuscript, and approved final manuscript. CS and AH contributed to data acquisition, significant contribution to manuscript, and approved final manuscript. RG performed data acquisition, data evaluation, significant contribution to manuscript, and approved final manuscript. All authors contributed to the article and approved the submitted version.

## Conflict of Interest

SN was a European Resuscitation Council—Instructor Educator Support Science and Education Committee (SEC-IES) member, and the current education representative of the young ERC group of the European Resuscitation Council. SN was also a Canadian Anesthesiologists’ Society (CAS) CEPD (Continuing Education and Professional Development) Committee member. RG was the European Resuscitation Council’s Board Director of Guidelines and ILCOR and Chair of the ILCOR Task Force on Education, Implementation, and Team. SN, CS, AH, and RG were currently Life Support instructors with the European Resuscitation Council. The remaining authors declare that the research was conducted in the absence of any commercial or financial relationships that could be construed as a potential conflict of interest.

## Publisher’s Note

All claims expressed in this article are solely those of the authors and do not necessarily represent those of their affiliated organizations, or those of the publisher, the editors and the reviewers. Any product that may be evaluated in this article, or claim that may be made by its manufacturer, is not guaranteed or endorsed by the publisher.

## Footnotes

1
https://www.randomizer.org

## References

[1] Sandroni C Nolan J Cavallaro F Antonelli M. In-hospital cardiac arrest: incidence, prognosis and possible measures to improve survival. *Intensive Care Med.* (2007) 33:237–45. 10.1007/s00134-006-0326-z 17019558

[2] Grasner JT Herlitz J Tjelmeland IBM Wnent J Masterson S Lilja G European resuscitation council guidelines 2021: epidemiology of cardiac arrest in Europe. *Resuscitation.* (2021) 161:61–79. 10.1016/j.resuscitation.2021.02.007 33773833

[3] Perkins GD Graesner JT Semeraro F Olasveengen T Soar J Lott C European resuscitation council guidelines 2021: executive summary. *Resuscitation.* (2021) 161:1–60. 10.1016/j.resuscitation.2021.02.003 33773824

[4] Smith GB Welch J DeVita MA Hillman KM Jones D. Education for cardiac arrest–treatment or prevention? *Resuscitation.* (2015) 92:59–62. 10.1016/j.resuscitation.2015.04.018 25921543

[5] Greif R Lockey A Breckwoldt J Carmona F Conaghan P Kuzovlev A European resuscitation council guidelines 2021: education for resuscitation. *Resuscitation.* (2021) 161:388–407. 10.1016/j.resuscitation.2021.02.016 33773831

[6] Nabecker S Huwendiek S Theiler L Huber M Petrowski K Greif R The effective group size for teaching cardiopulmonary resuscitation skills - A randomized controlled simulation trial. *Resuscitation.* (2021) 165:77–82. 34107336 10.1016/j.resuscitation.2021.05.034

[7] Lockey A Lin Y Cheng A. Impact of adult advanced cardiac life support course participation on patient outcomes-A systematic review and meta-analysis. *Resuscitation.* (2018) 129:48–54. 10.1016/j.resuscitation.2018.05.034 29902494

[8] Greif R Bhanji F Bigham BL Bray J Breckwoldt J Cheng A Education, implementation, and teams: 2020 international consensus on cardiopulmonary resuscitation and emergency cardiovascular care science with treatment recommendations. *Resuscitation.* (2020) 156:A188–239. 10.1016/j.resuscitation.2020.09.014 33098918

[9] Greif R Lockey AS Conaghan P Lippert A De Vries W Monsieurs KG European resuscitation council guidelines for resuscitation 2015: section 10. Education and implementation of resuscitation. *Resuscitation.* (2015) 95:288–301. 10.1016/j.resuscitation.2015.07.032 26477418

[10] Kuzovlev A Monsieurs KG Gilfoyle E Finn J Greif R Education Implementation and Teams Task Force of the International Liaison Committee on Resuscitation. The effect of team and leadership training of advanced life support providers on patient outcomes: a systematic review. *Resuscitation.* (2021) 160:126–39. 10.1016/j.resuscitation.2021.01.020 33556422

[11] Wyckoff MH Singletary EM Soar J Olasveengen TM Greif R Liley HG 2021 International Consensus on Cardiopulmonary Resuscitation and Emergency Cardiovascular Care Science With Treatment Recommendations: summary From the Basic Life Support; Advanced Life Support; Neonatal Life Support; Education, Implementation, and Teams; First Aid Task Forces; and the COVID-19 Working Group. *Resuscitation.* (2021) 69:229–311. 10.1016/j.resuscitation.2021.10.040 34933747 PMC8581280

[12] Fletcher G Flin R McGeorge P Glavin R Maran N Patey R. Anaesthetists’ non-technical skills (ANTS): evaluation of a behavioural marker system. *Br J Anaesth.* (2003) 90:580–8. 10.1093/bja/aeg112 12697584

[13] Gerstle CR. Parallels in safety between aviation and healthcare. *J Pediatr Surg.* (2018) 53:875–8. 10.1016/j.jpedsurg.2018.02.002 29506813

[14] Cooper S Cant R Porter J Sellick K Somers G Kinsman L Rating medical emergency teamwork performance: development of the team emergency assessment measure (TEAM). *Resuscitation.* (2010) 81:446–52. 10.1016/j.resuscitation.2009.11.027 20117874

[15] Mishra A Catchpole K McCulloch P. The Oxford NOTECHS System: reliability and validity of a tool for measuring teamwork behaviour in the operating theatre. *Qual Saf Health Care.* (2009) 18:104–8. 10.1136/qshc.2007.024760 19342523

[16] Malec JF Torsher LC Dunn WF Wiegmann DA Arnold JJ Brown DA The mayo high performance teamwork scale: reliability and validity for evaluating key crew resource management skills. *Simul Healthc.* (2007) 2:4–10. 10.1097/SIH.0b013e31802b68ee 19088602

[17] Moorthy K Munz Y Adams S Pandey V Darzi A. A human factors analysis of technical and team skills among surgical trainees during procedural simulations in a simulated operating theatre. *Ann Surg.* (2005) 242:631–9. 10.1097/01.sla.0000186298.79308.a8 16244534 PMC1409864

[18] Dagnone JD Hall AK Sebok-Syer S Klinger D Woolfrey K Davison C Competency-based simulation assessment of resuscitation skills in emergency medicine postgraduate trainees - a Canadian multi-centred study. *Can Med Educ J.* (2016) 7:e57–67. 27103954 PMC4830374

[19] Ringsted C Lippert F Hesselfeldt R Rasmussen MB Mogensen SS Frost T Assessment of Advanced Life Support competence when combining different test methods–reliability and validity. *Resuscitation.* (2007) 75:153–60. 10.1016/j.resuscitation.2007.03.003 17467869

[20] Boileau E Patenaude J St-Onge C. Twelve tips to avoid ethical pitfalls when recruiting students as subjects in medical education research. *Med Teach.* (2018) 40:20–5. 10.1080/0142159X.2017.1357805 28758523

[21] Walsh O Lydon S O’Connor P. A mixed methods evaluation of paediatric trainee preparedness to manage cardiopulmonary arrests. *Eur J Pediatr.* (2017) 176:1653–62. 10.1007/s00431-017-3017-6 28932935

[22] Manser T. Teamwork and patient safety in dynamic domains of healthcare: a review of the literature. *Acta Anaesthesiol Scand.* (2009) 53:143–51. 10.1111/j.1399-6576.2008.01717.x 19032571

[23] Norris EM Lockey AS. Human factors in resuscitation teaching. *Resuscitation.* (2012) 83:423–7. 10.1016/j.resuscitation.2011.11.001 22120456

[24] Hunziker S Johansson AC Tschan F Semmer NK Rock L Howell MD Teamwork and leadership in cardiopulmonary resuscitation. *J Am Coll Cardiol.* (2011) 57:2381–8. 10.1016/j.jacc.2011.03.017 21658557

[25] Fernandez Castelao E Boos M Ringer C Eich C Russo SG. Effect of CRM team leader training on team performance and leadership behavior in simulated cardiac arrest scenarios: a prospective, randomized, controlled study. *BMC Med Educ.* (2015) 15:116. 10.1186/s12909-015-0389-z 26205962 PMC4526177

[26] Peltonen V Peltonen LM Salantera S Hoppu S Elomaa J Pappila T An observational study of technical and non-technical skills in advanced life support in the clinical setting. *Resuscitation.* (2020) 153:162–8. 10.1016/j.resuscitation.2020.06.010 32561474

[27] Cooper S Wakelam A. Leadership of resuscitation teams: “Lighthouse Leadership’. *Resuscitation.* (1999) 42:27–45. 10.1016/s0300-9572(99)00080-5 10524729

[28] Cant RP Porter JE Cooper SJ Roberts K Wilson I Gartside C. Improving the non-technical skills of hospital medical emergency teams: the team emergency assessment measure (TEAM). *Emerg Med Australas.* (2016) 28:641–6. 10.1111/1742-6723.12643 27474369

[29] Andersen PO Jensen MK Lippert A Østergaard D. Identifying non-technical skills and barriers for improvement of teamwork in cardiac arrest teams. *Resuscitation.* (2010) 81:695–702. 10.1016/j.resuscitation.2010.01.024 20304547

[30] Jones CPL Fawker-Corbett J Groom P Morton B Lister C Mercer SJ. Human factors in preventing complications in anaesthesia: a systematic review. *Anaesthesia.* (2018) 73:12–24. 10.1111/anae.14136 29313908

[31] Ulmer FF Lutz AM Muller F Riva T Bütikofer L Greif R. Communication patterns during routine patient care in a pediatric intensive care unit: the behavioral impact of in situ simulation. *J Patient Saf.* (2021) 18:e573–9. 10.1097/PTS.0000000000000872 34224500

[32] Lee SH Khanuja HS Blanding RJ Sedgwick J Pressimone K Ficke JR Sustaining teamwork behaviors through reinforcement of TeamSTEPPS principles. *J Patient Saf.* (2017) 17:e582–6. 10.1097/PTS.0000000000000414 29087977

[33] Hunziker S Buhlmann C Tschan F Balestra G Legeret C Schumacher C Brief leadership instructions improve cardiopulmonary resuscitation in a high-fidelity simulation: a randomized controlled trial. *Crit Care Med.* (2010) 38:1086–91. 10.1097/CCM.0b013e3181cf7383 20124886

[34] Marx D Greif R Egloff M Balmer Y Nabecker S. Recruiting medical students for a first responder project in the social age: direct contact still outperforms social media. *Emerg Med Int.* (2020) 2020:9438560. 10.1155/2020/9438560 32566309 PMC7285391

[35] Nabecker S Theodorou M Huwendiek S Kasper N Greif R. Out-of-hospital cardiac arrest: comparing organised groups to individual first responders: a qualitative focus group study. *Eur J Anaesthesiol.* (2021) 38:1096–104. 10.1097/EJA.0000000000001335 33074938

