# Erratum: Assessment of human factors after advanced life support courses comparing simulated team and real team assessment: A randomised controlled cohort trial

**DOI:** 10.3389/fcvm.2023.1181500

**Published:** 2023-03-17

**Authors:** Frontiers Production Office

**Affiliations:** Frontiers Media SA, Lausanne, Switzerland

**Keywords:** education, CPR, life support, European resuscitation council (ERC), human factors

An Erratum on Assessment of human factors after advanced life support courses comparing simulated team and real team assessment: A randomized controlled cohort trial By Nabecker S, Huwendiek S, Seidl C, Hana A, Theiler L and Greif R (2022) Front. Cardiovasc. Med. 9:840114. doi: 10.3389/fcvm.2022.840114

An omission to the funding section of the original article was made in error. The following sentence has been added: “Open access funding was provided by the University of Bern”.

The original version of this article has been updated.

